# DKI and ^1^H-MRS in angiogenesis evaluation of soft tissue sarcomas: a prospective clinical study based on MRI-pathology control method

**DOI:** 10.1186/s12880-024-01526-8

**Published:** 2024-12-18

**Authors:** Wubing Han, Cheng Xin, Zeguo Wang, Fei Wang, Yu Cheng, Xingrong Yang, Yangyun Zhou, Juntong Liu, Wanjiang Yu, Shaowu Wang

**Affiliations:** 1 Department of Radiology, Qingdao Municipal Hospital, University of Health and Rehabilitation Sciences, 5 Donghai Middle Rd, Qingdao, 266071 China; 2 Department of Radiology, The Second Hospital, Dalian Medical University, 467 Zhongshan Rd, Dalian, 116023 China; 3 Department of Pathology, The Second Hospital, Dalian Medical University, 467 Zhongshan Rd, Dalian, 116023, China

**Keywords:** Diffusion kurtosis imaging, Proton magnetic resonance spectroscopy, Vascular endothelial growth factor, Microvessel density, Soft tissue sarcoma

## Abstract

**Background:**

The vascular endothelial growth factor (VEGF) and microvessel density (MVD) have been widely employed as angiogenesis indicators in the diagnosis and treatment of soft tissue sarcomas. While diffusion kurtosis imaging (DKI) and proton magnetic resonance spectroscopy (^1^H-MRS) imaging hold potential in assessing angiogenesis in other tumors, their reliability in correlating with angiogenesis in soft tissue sarcomas remains uncertain, contingent upon accurately acquiring the region of interest (ROI).

**Methods:**

23 patients with soft tissue sarcomas (STSs) confirmed by pathology were selected, underwent DKI and ^1^H-MRS at 3.0T MRI. The DKI parameters mean diffusivity (MD), mean kurtosis (MK), kurtosis anisotropy (KA), and ^1^H-MRS parameters choline (Cho), lipid/lactate (LL) were measured by two radiologists. Two pathologists obtained pathological slices using a new sampling method called MRI-pathology control and evaluated VEGF and MVD in the selected regions. Correlations between MRI parameters and angiogenesis markers were assessed by Person or Spearman tests.

**Results:**

The DKI parameters MD and KA, and the ^1^H-MRS parameters Cho and LL, have varying degrees of correlation with the expression levels of VEGF and MVD. Among them, Cho exhibits the strongest correlation (*r* = 0.875, *P* < 0.001; *r* = 0.807, *P* < 0.001).

**Conclusion:**

Based on this preliminary clinical studies, DKI and ^1^H-MRS parameters are correlated with angiogenesis markers obtained through the “MRI-pathology control” method.

## Introduction

Soft Tissue Sarcomas (STSs) are a group of tumors originating from mesenchymal tissues, account for less than 1% of all malignant tumors. Despite their low incidence, they are characterized by high malignancy and poor prognosis [1, 2]. Vascular Endothelial Growth Factor (VEGF) and Microvessel Density (MVD) significantly influence the growth, metastasis, and prognosis of STSs [3]; their high expression can predict tumor progression and sensitivity to anti-angiogenic therapy [4–6]. However, the assessment of VEGF and MVD requires invasive pathological sampling, which cannot be performed repeatedly, limiting their clinical application [7].

Magnetic Resonance Imaging (MRI) is a non-invasive tool for evaluating STSs, providing anatomical and biological information through functional imaging techniques [8]. Diffusion Kurtosis Imaging (DKI) more accurately characterizes tissue microstructure compared to traditional DWI [9, 10]; Proton Magnetic Resonance Spectroscopy (¹H-MRS) obtains the spatial distribution and relative concentrations of different chemical components in tissues [11, 12]. These advanced imaging techniques hold promise for the non-invasive assessment of angiogenesis in STSs, guiding the formulation of individualized treatment plans and improving therapeutic outcomes.

Currently, there are few non-invasive predictive studies on angiogenesis in human extremity STSs, and most focus on DWI, lacking systematic research on DKI and ¹H-MRS [13–17]. This study aims to assess the angiogenesis of STSs based on the MRI-pathology control method, combining DKI and ¹H-MRS techniques. By filling this research gap, we hope to provide reliable imaging indicators for clinical practice to guide anti-angiogenic therapy.

## Materials and methods

### Patients

**Fig. 1:**
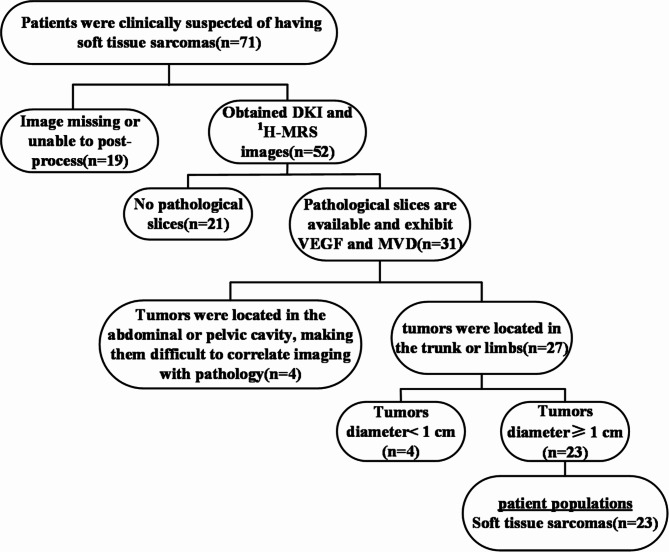
Flowchart of patient inclusion criteria

The Ethics Committee of The Second Hospital of Dalian Medical University gave autorization for all experiments(NO:2022053). A total of 71 patients suspected of STS were continuously collected from September 2020 to October 2023. These patients underwent preoperative MRI, including DKI and ¹H-MRS. After applying exclusion criteria, 23 patients with STSs remained in the study group, there were 17 males (mean age 53) and 6 females(mean age 52). The pathological type of the patients can be found in Table 1. The exclusion criteria were: (1) missing or difficult-to-analyze MRI sequences due to unclear images; (2) absence of pathological sections; (3) tumor site not suitable for the imaging-pathology control method; (4) MRI findings indicating that the maximum diameter of the tumor tissues was < 1.0 cm. (Fig. 1)

**Table 1:** The pathological type of soft tissue sarcoma

Histological types	Total	Tumor size (cm)
dedifferentiated liposarcoma	1	16.00
myxoid liposarcoma	4	17.61 ± 7.96
sclerosing liposarcoma	1	14.33
well-differentiated liposarcoma	1	12.10
alveolar rhabdomyosarcoma	1	10.20
pleomorphic rhabdomyosarcoma	1	7.33
undifferentiated pleomorphic sarcoma	3	4.61 ± 1.85
dermatofibrosarcoma protuberans	1	3.50
epithelioid leiomyosarcoma	1	4.42
myxoid leiomyosarcoma	2	11.95 ± 2.25
inflammatory leiomyosarcoma	1	4.10
synovial sarcoma	5	11.49 ± 3.66
fibrosarcoma	1	9.40

### MRI protocol

All MRI scans were performed on a 3.0T (Discovery 750w, GE Healthcare) whole body scanner using a 16-channel phased-array abdominal coil for the transmit-receive coil. Conventional MRI was performed to determine the tumor’s location, including axial T1WI, axial T2WI, coronal T2WI, and sagittal T2WI, and corresponding fat-suppressed images. DKI and ¹H-MRS were performed after the conventional MRI, with b-values of 0, 1000, and 2000 s/mm² for the DKI sequence, and 15 diffusion gradient directions applied for each b-value. The solid component of the tumor (excluding liquefied and necrotic areas), as evaluated by the conventional MRI, was used as the scanning area for ¹H-MRS. Saturation bands were employed to shield interfering signals such as water, bone, blood vessels, and nerves around the lesion, with a voxel size of 2 × 2 × 2 mm. The parameters for each scanning sequence are shown in Table 2.

**Table 2:** Details of MRI parameters

	Sequence	TR(ms)	TE(ms)	NEX	Martrix
Axi-T1WI	FSE	478 ∼ 667	13 ∼ 19	2	512 × 512
Cor-T1WI	FSE	478 ∼ 689	15 ∼ 19	2	512 × 512
Sag-T1WI	FSE	478 ∼ 689	15 ∼ 19	2	512 × 512
Axi-T2WI	FSE	3063 ∼ 4940	70 ∼ 96	2	512 × 512
Cor-T2WI	FSE	3075 ∼ 4500	67 ∼ 109	2	512 × 512
Sag-T2WI	FSE	3075 ∼ 4500	67 ∼ 109	2	512 × 512
DKI	SE-EPI	3000	93	3	128 × 128
^1^H-MRS	PRESS	1500	144	1	128 × 128

*Note*: *TR* repetition time; *TE* echo time; *NEX* number of excitation; *Axi* axial; *Cor* coronal; *Sag* sagittal; *T1WI* T1-weighted imaging; *T2WI* T2-weighted imaging; *DKI* diffusion kurtosis imaging; ^*1*^*H-MRS* proton magnetic resonance spectroscopy; *FSE* fast spin echo; *SE-EPI* spin echo- echoplanar imaging; *PRESS* point resolved spectroscopy

### Quantitative assessment of MRI

DKI and ¹H-MRS images were transmitted to a GE Advantage Workstation 4.7, and the raw images were processed using Function Tool software to generate corresponding pseudo-color images. ROIs were selected by two radiologists with over 10 years of experience in musculoskeletal radiology. The DKI sequence was used to outline ROIs for the lesion area according to the following principles: (1) conventional MRI were referenced to select the significantly enhanced part of the tumor while avoiding cystic lesions; (2) the size of the area of interest was 50–60 mm²; (3) thresholds were adjusted to obtain Mean Diffusivity (MD), Mean Kurtosis (MK), and Kurtosis Anisotropy (KA) values, and generate the corresponding pseudo-color images. The ROI from the same region in DKI was employed to determine the mean voxel value in the corresponding region of ¹H-MRS, facilitating the determination of Choline (Cho) and Lipid/Lactate (LL) values.

### MRI-pathology control method

**Fig. 2:**
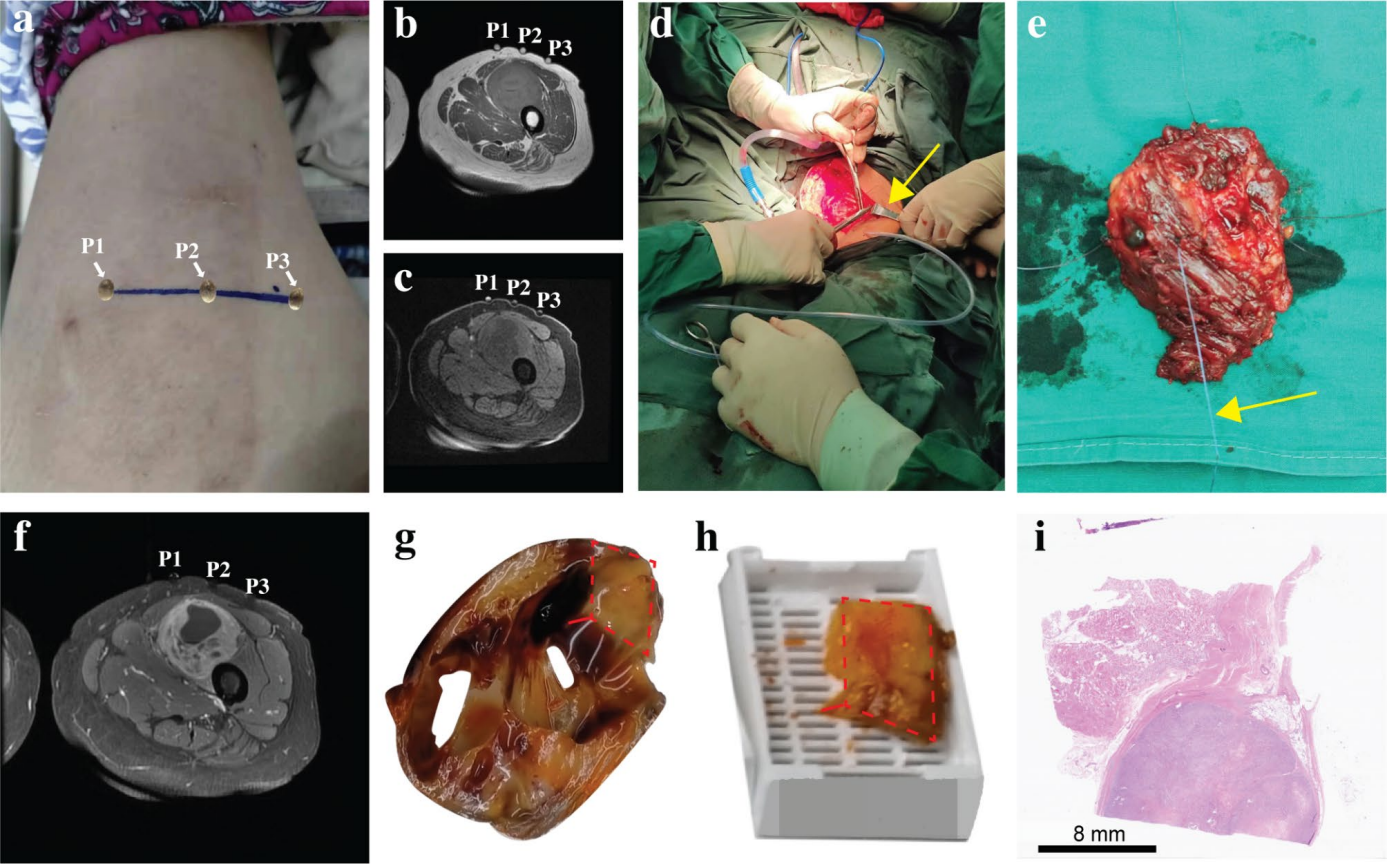
MRI-Pathology control method

To improve the accuracy of imaging and pathology in the assessment of soft tissue sarcomas, a method called MRI-pathology level control was applied [13, 14]. Refer to Fig. 2 for specific steps:

1. Imaging level with pathology sampling control: The maximum diameter of the tumor was determined through palpation on the patient’s body surface, marking it with a line at the most prominent place, and placing gelatin capsules P1, P2, and P3 on the line at equal intervals (Fig. 2a). The patient underwent MRI, and the level where the three gelatin capsules appeared simultaneously was designated as the control level (Fig. 2b, Fig. 2c). During surgery, the patient’s tumor was exposed and then fixed with a white wire to determine the maximal diameter of the mass (Fig. 2d, Fig. 2e).
2. Imaging ROI with pathology section control: The solid component of the tumor at the largest level was identified based on the enhanced T1WI image (Fig. 2f). The tumor was incised along the white line to obtain the largest level (Fig. 2g), and the solid area corresponding to the enhanced T1WI was used to produce the pathology section (Fig. 2 h, Fig. 2i).

### Histological analysis

Based on the MRI-pathology control method, the pathology level corresponding to the imaging level was selected to make pathology sections, which were stained with H&E, followed by immunohistochemical staining for CD34 and VEGF. Two pathologists with over 10 years of experience in soft tissue tumor pathology confirmed the 23 cases into STSs according to the World Health Organization’s STS diagnostic criteria in 2020 [18], and scored the VEGF using a point system. Following the method of Guo et al. [19], the percentage of stained cells among the total number of cells in the high-power microscope (×400) field of view was used as a classification criterion, dividing the samples into four grades: negative, ≤ 10%, 11-50%, and 51-75%, with corresponding scores of 0, 1, 2, 3, and 4 points, respectively. The staining intensity was divided into four grades: non-staining, yellowish, brownish-yellow, and brownish-brown, with corresponding scores of 0, 1, 2, and 3 points. The VEGF score was calculated as the sum of the intensity scores and the percentage of positive cells across five fields of view. The average VEGF expression score across different fields of view was taken as the final result. MVD values were scored using a positive cell counting system, referencing the method of Weidner et al. [20]. The whole section was first observed under low magnification (×40) to identify areas where microvessels were concentrated, followed by counting the number of labeled microvessels in three fields of view under high magnification (×200). Counting criteria included all endothelial cells or vascular endothelial cells, regardless of the presence of a lumen, stained brownish-yellow and considered as one independent microvessel, while large vessels with a diameter greater than 8 erythrocyte diameters or with a thick smooth muscle layer were excluded. The average of the three fields of view was taken as the MVD value of the specimen.

### Statistical analysis

The analysis of all data was performed using SPSS 24.0 (IBM Corp., Chicago, IL, USA), with all data presented as mean ± standard deviation (SD). Interclass correlation coefficients (ICCs) were employed to assess the consistency of parameter measurements between two sets of observers within each group (radiological and pathological). The Kolmogorov-Smirnov test was employed to analyze whether the data conformed to a normal distribution (*P* < 0.05 indicating non-normal distribution). When radiological parameters (MD, MK, KA, Cho, LL) and pathological parameters (VEGF, MVD) conform to a normal distribution, the Pearson correlation test is utilized; otherwise, the Spearman correlation test is employed.

## Results

### Interobserver agreement

The Inter-observer consistency between the two radiologists for the measurement of imaging parameters (MD, MK, KA, Cho, LL) and pathology parameters (VEGF, MVD) was good. ICC values ranged from 0.855 to 0.944, indicating good reproducibility. (Table 3)

**Table 3:** Inter-observer consistency test

parameter	ICC	95% Confidence Interval	*P*
MD	0.913	0.844–0.962	< 0.01
MK	0.855	0.830–0.871	< 0.01
KA	0.944	0.919–0.975	< 0.01
Cho	0.923	0.917–0.934	< 0.01
LL	0.899	0.878–0.908	< 0.01
VEGF	0.935	0.912–0.958	< 0.01
MVD	0.908	0.881–0.924	< 0.01

*Note: ICC* intra-class correlation coefficient; *95%CI* confidence interval; *MD* mean diffusivity; *MK* mean kurtosis; *KA* kurtosis anisotropy; *Cho* choline; *LL* Lipid/Lactate; *VEGF* vascular endothelial growth factor; *MVD* microvessel density

### Parameters derived from MRI and pathology

**Fig. 3:**
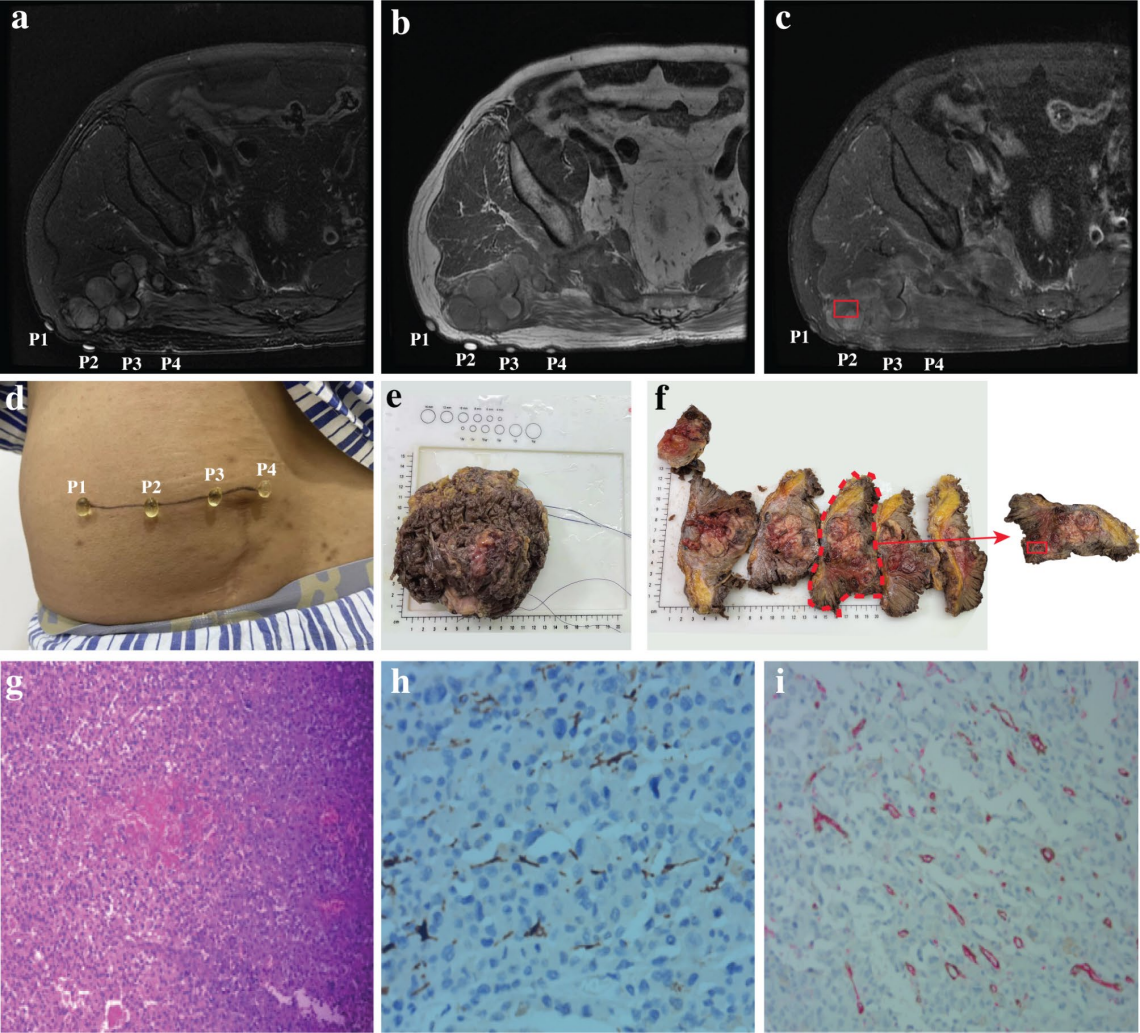
A patient with pleomorphic rhabdomyosarcoma in right buttock. (**a**) Axial T2-weighted images showed a cystic-solid mass presenting with an irregular high signal. (**b**) Axial T1-weighted images showed that the mass is mostly low signal with a few high signal hemorrhagic foci. (**c**) The enhanced axial T1-weighted images showed that heterogeneous enhancement of the solid components of the mass, while the cystic components do not enhance, confirming that the red box was the pathological sampling area. (**d-e**) The marking of the tumor’s largest diameter on the patient’s body surface (four capsules), and the marking of the tumor’s largest diameter after surgery (black lines). (**f**) Selection of pathological sampling points (based on enhanced axial T1-weighted images). (**g**) H&E(×100). (**h**) VEGF(×400). (**i**) MVD(×200)

**Fig. 4:**
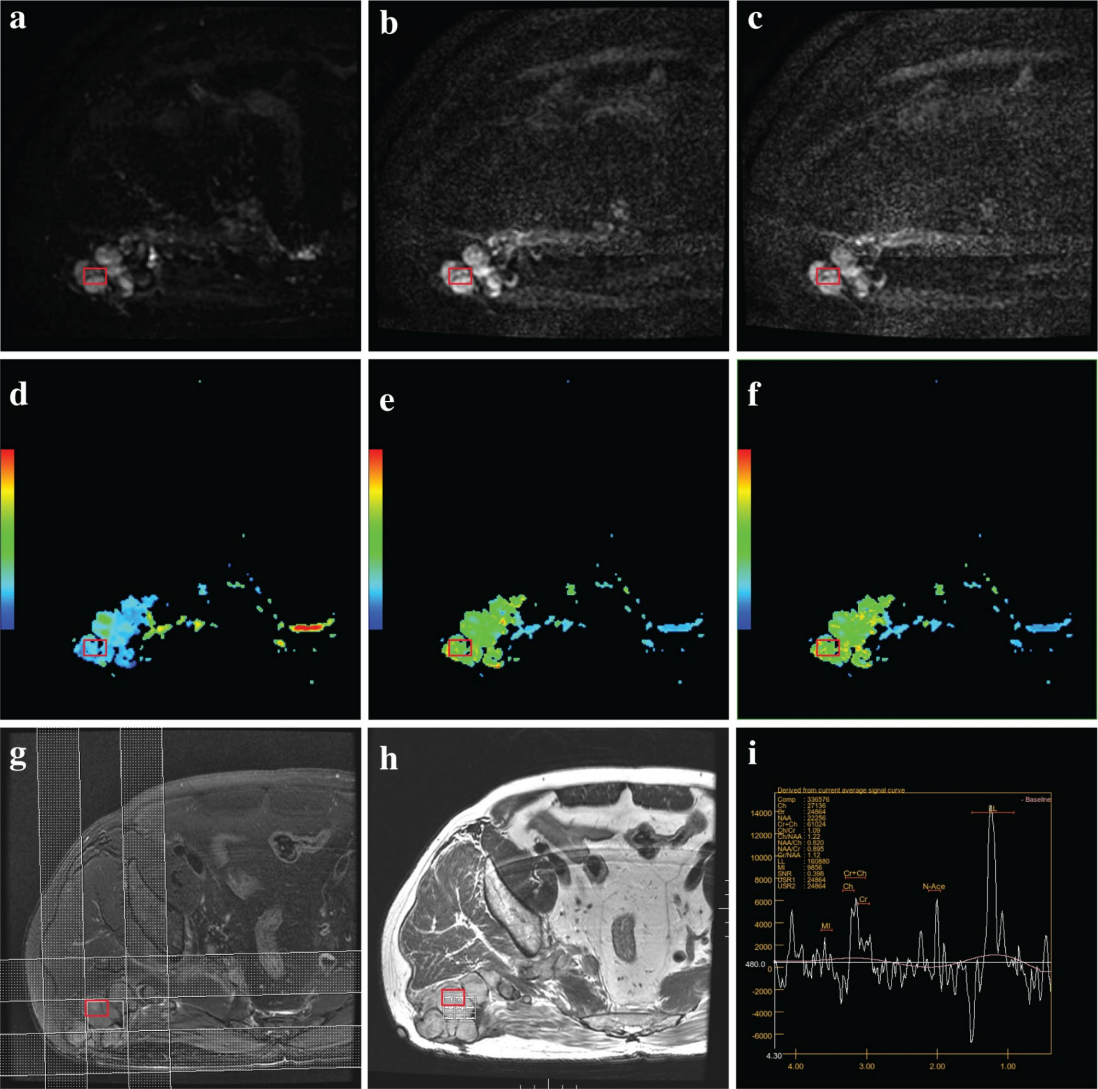
A patient with pleomorphic rhabdomyosarcoma in right buttock, the red box indicates the region of interest. (**a-c**) DKI showed the tumor exhibiting a high signal (b = 0,1000,2000 s/mm2). (**d-f**) The post-processing images for the tumor’s MD, MK, and KA were displayed in blue, light green, and yellow-green, respectively. (**g-i**) Post-processed ^1^H-MRS images showed that the Cho and LL peaks significantly elevated in the tumor

Refer to Fig. 3 and Fig. 4 for the acquisition of MRI and pathology parameters. VEGF score was 3.12 ± 2.31 (ranged from 0.4 to 10.10) and MVD value was 139.88 ± 96.61 (ranged from 15.30 to 366.70). The values of the DKI parameters MD, MK, and KA were 1.62 ± 0.91 (ranged from 0.21 to 3.53), 0.83 ± 0.47 (ranged from 0.24 to 2.11), and 0.87 ± 0.53 (ranged from 0.22 to 2.52) respectively. The values of the ¹H-MRS parameters Cho and LL were 12.05 ± 10.96 (ranged from 1.03 to 49.41) and 194.13 ± 136.73 (ranged from 21.32 to 677.00).

### Correlation of DKI and 1H-MRS with VEGF and MVD expression

**Fig. 5:**
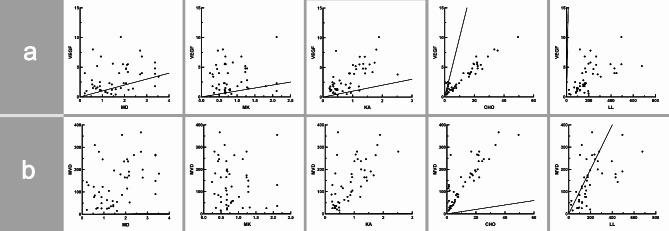
(**a**) As shown in the scatter plot, the correlation between DKI parameters MD, MK, KA, and ^1^H-MRS parameters Cho, LL with VEGF. (**b**) The correlation between DKI parameters MD, MK, KA, and ^1^H-MRS parameters Cho and LL with MVD

Cho was strongly positively correlated with VEGF and MVD (*r* = 0.875, *P* < 0.01; *r* = 0.801, *P* < 0.01); KA was moderately positively correlated with VEGF and MVD (*r* = 0.632, *P* < 0.001; *r* = 0.674, *P* < 0.001); LL was moderately positively correlated with VEGF and MVD (*r* = 0.696, *P* < 0.001; *r* = 0.676, *P* < 0.001); MD was weakly positively correlated with VEGF and MVD (*r* = 0.369, *P* = 0.08; *r* = 0.448, *P* < 0.001); and MK was not correlated with VEGF and MVD (*r* = 0.055, *P* = 0.710; *r*=-0.147, *P* = 0.315). (Fig. 5)

## Discussion

In this study, metabolic imaging sequence ¹H-MRS was applied for the first time to reflect angiogenesis based on the diffusion sequence DKI. The MRI-pathology control method was employed to ensure that pathology section points accurately corresponded to the image ROIs. The results indicated that MD, LL, and KA had significant potential in reflecting angiogenesis in STSs.

VEGF is present in vascular endothelial cells and is the most crucial factor in regulating angiogenesis. In normal tissues, VEGF is in a quiescent state and is activated in large quantities only in damaged or pathological tissues. Tumor tissue secretes a large amount of VEGF, leading to the formation of numerous new blood vessels within the tumor, which results in the rapid proliferation of tumor tissues [21–23]. MVD is an immunohistochemical counting method used to quantitatively assess the microvessel density of tissues. The metabolic proliferation level of tumor tissues is higher than that of normal tissues, necessitating more blood vessels to supply nutrients. Consequently, the microvessel density of tumor tissues is higher than that of normal tissues [24, 25]. Several studies have demonstrated the value of VEGF and MVD in differentiating benign and malignant soft tissue tumors, identifying subtypes, and predicting recurrence and metastasis.

DKI is one of the commonly used sequences for diffusion-weighted imaging, introducing the concept of kurtosis to quantify the non-Gaussian movement of water molecules. It has been shown to reflect the degree of diffusion limitation of water molecules in tissues more accurately than DWI, DTI, and IVIM. The main parameters of DKI are MD, MK, and KA, with higher values indicating more complex tissue structures [26]. In previous studies, MD and MK have been used to determine border invasion, assess cellular accretion, and evaluate hypoxia in STSs. To the best of our knowledge, this is the first study to use DKI to assess angiogenesis in STSs [16, 27, 28]. To the best of our knowledge, this is the first study to use DKI to assess angiogenesis in STSs. The results showed that MD was positively correlated with both VEGF and MVD, but not with MK values. This differs from the results of some other studies [29], and the reasons for this weak correlation may include the following aspects. Firstly, STSs are highly heterogeneous, with varying histological types, cell densities, and matrix components. The MK value reflects the non-Gaussian diffusion of water molecules in tissues and is influenced by the cellular microenvironment. In tissues with high heterogeneity, the MK value may be affected by multiple factors, reducing its correlation with angiogenesis indicators [30]. Secondly, the impact of the tumor microenvironment cannot be ignored. Common pathological changes in STSs, such as necrosis, hemorrhage, and calcification, may affect the diffusion behavior of water molecules. The MK value is sensitive to these microstructural changes, but these changes may not be directly related to VEGF and MVD, thereby weakening the correlation [31]. This study confirmed that STSs exhibit robust angiogenesis and tightly packed cellular arrangements [Fig. 3 h, 3i], leading to elevated MD values. In previous research, KA has been primarily used to diagnose central nervous system diseases such as brain glioma [32], cerebral vascular malformations [33], and Alzheimer’s disease [34]. In our study, KA was used for the first time in soft tissue sarcomas, with the results indicating a strong correlation between KA and angiogenesis, which was stronger than the correlation with MD values.

¹H-MRS is a sequence in medical magnetic resonance imaging that can evaluate the metabolism of living tissues. The quantitative parameters related to soft tissues in ¹H-MRS include choline (Cho), lactic acid (Lac), and lipid (Lipid). Cho is a marker of cellular membrane activity, with its concentration increasing alongside cell density. Lac/Lipid reflects the degree of hypoxic necrosis in tissue, with the appearance of the Lac/Lipid peak indicating inhibited aerobic respiration and enhanced glycolytic processes [35–37]. Our study found that Cho and LL were significantly positively correlated with VEGF and MVD in soft tissue sarcoma, differing from the findings of Jansen et al. [38]. However, it was confirmed that the exuberant angiogenesis in soft tissue sarcomas leads to over-activation of the cell membrane, resulting in an elevated Cho peak. Exuberant angiogenesis, the excessive formation of new blood vessels, is a hallmark of soft tissue sarcomas. This heightened angiogenic activity can lead to over-activation of the cell membrane, which in turn can result in an elevated Cho peak. This phenomenon is often observed in MRS or MRI studies of soft tissue sarcomas. The elevated Cho peak can serve as a biomarker indicating increased cell turnover and membrane synthesis, reflecting the aggressive nature of the tumor. Understanding these molecular changes can be crucial for diagnosis, treatment planning, and monitoring the response to therapy in patients with soft tissue sarcomas [39]. Additionally, the overgrowing tumor center results in hypoxic necrosis, leading to an elevated LL peak [40]. It should be noted that our study employed the MRI-pathology control method, ensuring more precise image ROIs and pathology sections compared to previous studies.

Our study has several limitations. Firstly, the low incidence and challenging follow-up of STSs have resulted in our limited sample size. Secondly, there may be selection bias in our sample. We included cases mainly comprising STS subtypes that are difficult to diagnose using conventional MRI sequences, and excluded vascular tumors because they may lead to abnormally elevated levels of VEGF and MVD, potentially introducing bias into the study. This selection may limit the external validity of the study results and may not fully reflect the applicability of DKI and ¹H-MRS across all types of soft tissue sarcomas. Thirdly, limitations of the MRI-pathology control method. This method is only applicable to soft tissue sarcomas in the limbs and superficial areas and requires a tumor diameter of ≥ 1 cm. Therefore, retroperitoneal and intra-abdominal STSs were excluded due to the difficulty in obtaining samples, which limits the application of our method to tumors in these locations. Furthermore, there are technical challenges in imaging and pathological correlation for deep-seated and small-volume tumors, which may affect the widespread adoption of this method. In future studies, we encourage conducting multicenter studies to expand the sample size and diversity. Additionally, we will create detailed flowcharts and develop standard operating procedures that cover every step from MRI scanning parameter settings to pathological slice preparation and staining, ensuring that all participants adhere to uniform operational standards. We will regularly calibrate MRI equipment to ensure data consistency across different devices, establish calibration standards and frequencies to reduce variability between devices, and standardize the thickness and staining methods of pathological slices (such as H&E staining, CD34, and VEGF immunohistochemical staining) to ensure consistency among different samples.

In conclusion, based on the MRI-pathology control method, DKI and ¹H-MRS correlate with VEGF and MVD in STSs, with Cho showing the strongest correlation, this indicates that DKI and ¹H-MRS has the potential to guide clinical treatment decisions, especially in the selection of anti-angiogenic therapy regimens. Although the MRI-pathology control method is a precise technique for tumor sampling, further simplification is needed for its application in future clinical practice.

## Data Availability

The datasets generated during the current study are not publicly available due Patient information confidentiality but are available from the corresponding author on reasonable request.

## Abbreviations

DKI: Diffusion kurtosis imaging
^1^H-MRS: Proton magnetic resonance spectroscopy
MD: Mean diffusivity
MK: Mean kurtosis
KA: Kurtosis anisotropy
Cho: Choline
LL: Lipid/Lactate
VEGF: Vascular endothelial growth factor
MVD: Microvessel density
STS: Soft tissue sarcoma
